# HLA Typing for the Next Generation

**DOI:** 10.1371/journal.pone.0127153

**Published:** 2015-05-27

**Authors:** Neema P. Mayor, James Robinson, Alasdair J. M. McWhinnie, Swati Ranade, Kevin Eng, William Midwinter, Will P. Bultitude, Chen-Shan Chin, Brett Bowman, Patrick Marks, Henny Braund, J. Alejandro Madrigal, Katy Latham, Steven G. E. Marsh

**Affiliations:** 1 Anthony Nolan Research Institute, Royal Free Hospital, London, United Kingdom; 2 UCL Cancer Institute, Royal Free Campus, London, United Kingdom; 3 Pacific Biosciences, Menlo Park, California, United States of America

## Abstract

Allele-level resolution data at primary HLA typing is the ideal for most histocompatibility testing laboratories. Many high-throughput molecular HLA typing approaches are unable to determine the phase of observed DNA sequence polymorphisms, leading to ambiguous results. The use of higher resolution methods is often restricted due to cost and time limitations. Here we report on the feasibility of using Pacific Biosciences’ Single Molecule Real-Time (SMRT) DNA sequencing technology for high-resolution and high-throughput HLA typing. Seven DNA samples were typed for HLA-A, -B and -C. The results showed that SMRT DNA sequencing technology was able to generate sequences that spanned entire HLA Class I genes that allowed for accurate allele calling. Eight novel genomic HLA class I sequences were identified, four were novel alleles, three were confirmed as genomic sequence extensions and one corrected an existing genomic reference sequence. This method has the potential to revolutionize the field of HLA typing. The clinical impact of achieving this level of resolution HLA typing data is likely to considerable, particularly in applications such as organ and blood stem cell transplantation where matching donors and recipients for their HLA is of utmost importance.

## Introduction

The HLA genes are located within one of the most gene rich regions of the human genome, the Major Histocompatibility Complex (MHC), on the short arm of chromosome 6 (6p21.3). Many of these genes, including HLA, encode proteins that have a critical role in immune responses[1, 2]. The MHC is divided into three distinct regions referred to as class I, II and III, with the HLA genes being located within the class I and class II regions. The HLA genes are known to be the most polymorphic genes of the human genome[1, 3]. This polymorphism is predominantly found within the six classical HLA genes: the class I genes HLA-A,-B and-C and the class II genes HLA-DRB1,-DQB1 and -DPB1. Over 12,200 HLA alleles have been identified to date (December 2014), with in excess of 9,200 being variants of the HLA class I genes alone [www.ebi.ac.uk/imgt/hla] [4, 5].

HLA proteins function as antigen presentation molecules presenting self and non-self peptides to T-cells, a fundamental step in the initiation of certain adaptive immune responses. Much of the described polymorphism within the HLA class I genes is located within the exons that encode the peptide binding groove and the points at which T-cells interact with the molecule itself. This diversity has evolved as a mechanism to ensure on-going pathogen recognition and eradication by increasing the repertoire of peptide motifs that can be bound and presented to T cells [6, 7]. Over-dominant selection is also thought to have driven the extent of polymorphism. HLA heterozygosity is favoured in a population because it increases the number of peptide motifs that can be presented by the co-dominantly expressed HLA molecules[7]. This strong heterozygote advantage is of particular importance in the event of infection by a pathogen that is specifically able to evade presentation by a particular HLA allele by ensuring that an individual is capable of initiating immune responses against the pathogen by presentation of the peptide by the second allele.

As the majority of described polymorphisms are located within the peptide binding groove that is encoded by exons 2 and 3 of the HLA class I genes, and that these differences have such an important functional relevance, many of the routinely used high-throughput HLA typing methods are focused on identifying variation within this limited region. A common problem encountered is the inability to determine the phase of polymorphisms identified in a single individual, a problem that is exacerbated by the extensive genetic diversity seen in HLA genes [8, 9]. The result of this is ambiguous HLA types and the reporting of HLA typing strings. The high workload, cost and time required to generate true allele-level HLA typing using current methods makes it preclusive for most histocompatibility laboratories.

The recent development of second-generation sequencing methods has been of great interest to the HLA typing community due to the possibility of sequencing a single DNA strand in isolation. These techniques provide an opportunity for single allele definition at primary HLA typing as opposed to cross-referencing results from different molecular techniques and serological testing. Previously, sequencing an entire HLA gene in isolation was achieved through the use of PCR-cloning processes, which are lengthy and often problematic. Second-generation sequencing methods have the potential to negate the use of such challenging laboratory practices. These technologies offered the first realistic solution to the problem of phasing polymorphisms throughout the HLA gene, enabling definitive allele typing. Consequently, many second-generation technologies have now been optimised for use by the HLA typing market and use of Sequence-Based Typing (SBT) protocols are common [10–14]. A current limitation of these methods are the read lengths that can be generated, resulting in the need for multiple over-lapping sequences to achieve full gene and even partial gene sequencing. A common concern with these methodologies is that incorrectly aligned fragments could result in HLA typing errors. It is possible that in a system as polymorphic as the HLA genes, incorrect phasing of SNPs that are distant to each other across the gene but otherwise show complete homology could result in an incorrect allele being assigned. Additionally rare or novel allele formed by a recombination event may be missed if the consensus sequence analysis tools are biased towards the more common alleles.

The ideal solution to resolve both HLA ambiguity and the potential problems caused by phasing multiple fragments would be to produce multiple long sequence reads encompassing whole gene PCR amplicons, in isolation. The development of Pacific Biosciences’ Single Molecule Real Time (SMRT) DNA sequencing technology offers the first realistic option to achieve this goal [15]. The SMRT sequencing method is able to generate exceptionally long read lengths that would allow coverage of the 3 kb or more of a HLA class I gene sequence, and thus determine the phase of the resolving polymorphisms seen. In addition, the technology has the potential to sequence read length in excess of 20 kb that could allow for entire HLA class II gene sequencing, which at over 10 kb for some genes, are substantially longer in length than the HLA class I genes.

**Fig 1:**
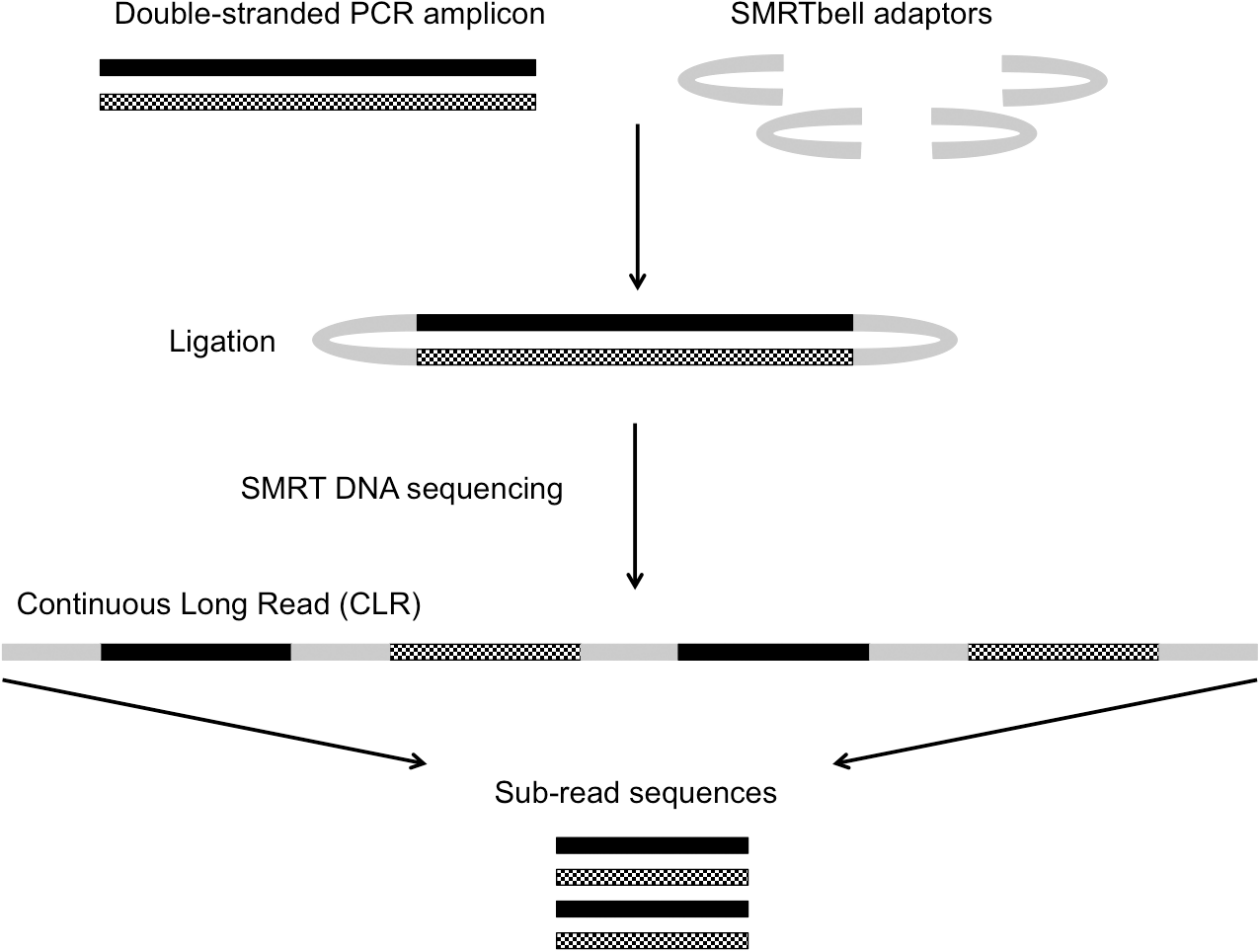
Basic stages of the Single Molecule Real-Time (SMRT) DNA sequencing method. SMRTbell adaptors are ligated onto the ends of a blunt-ended PCR amplicon to facilitate continuous sequencing of both strands of the amplicon. The entire sequence generated may include multiple copies of the sense and anti-sense strands of the PCR amplicon in a single read known as the Continuous Long Read (CLR). The post-sequencing bioinformatic post-processes are able to break down the CLR into shorter sub-reads, which encompass the sequence of one strand of the amplicon. These sub-reads can then be compared and used to create a consensus sequence.

SMRT DNA sequencing makes use of SMRTbell templates, single stranded hairpin adaptors that can be ligated on to the ends of PCR products. The function of these adaptors is to turn an essentially linear PCR amplicon into a circular molecule. The advantage of generating a circular molecule is that the enzyme added to facilitate the reaction is capable of processively generating sequence from both strands of the PCR amplicon until either the enzyme expires or the end of the run-time is achieved. Under optimal experimental conditions, the result of the continuous sequencing process is the generation of a Continuous Long Read (CLR); one exceptionally long read which contains multiple regions of sequence specific to the PCR amplicon (known as sub-reads) interspersed with the sequence of the SMRTbell adaptors (Fig 1). This novel method of generating DNA sequence means that it is possible to interrogate the same DNA strand multiple times within a single experiment, achieving exceptionally high depth of sequence coverage.

Here we describe the results of a study to determine whether the SMRT DNA sequencing methodology could be adapted for use in the Anthony Nolan Histocompatibility Laboratory to facilitate stem cell donor registry typing. The aims of this study were fourfold; i) to determine whether the methods were suitable for adaptation with Anthony Nolan DNA samples and PCR amplicons; ii) to determine if basic levels of multiplexing were possible; iii) to see if genomic HLA class I sequences could be generated; and iv) to determine the accuracy and specificity of the sequences generated.

## Material and Methods

Seven DNA samples were selected for HLA class I genotyping using Pacific Biosciences’ SMRT sequencing methodology. In recent years, Anthony Nolan has changed from blood to Oragene saliva (DNA Genotek, Ottawa, Canada) as their primary source of DNA when recruiting donors to our stem cell donor register and thus DNA from this source makes up a large part of our workload. In accordance with our in-house protocol, blood samples are still requested from donors who are short-listed as potential matches for confirmatory HLA typing, virology and other associated tests. To ensure that DNA from both starting materials were suitable for use with SMRT sequencing methods, two samples were selected from each category. Written consent for HLA typing was obtained from all blood and saliva sample donors at the point of collection for the purposes of transplantation. These consent forms are in accordance with the Human Tissue Authority (HTA) UK, European Federation for Immunogenetics (EFI), and Clinical Pathology Accreditation (CPA) regulatory body guidelines. Anthony Nolan’s Medical Advisory committee has also reviewed and approved the consent form. Specific approval from the local ethics committee was not sought, as the purpose of the study is to assess the method of HLA typing in comparison with existing HLA typing techniques. No new genetic information outside of the HLA genes that would affect the donors of the material used in this study has been gained.

Three B-Lymphoblastoid Cell Lines (B-LCLs) were also selected from a well-characterized panel that have been extensively analysed for their HLA genes. HLA class I genotyping was undertaken for all donor-derived DNA samples using Luminex LABType SSO typing kits (One Lambda, CA, USA). HLA genotype information for the B-LCLs was obtained from the IMGT/HLA Database website [www.ebi.ac.uk/ipd/imgt/hla/][8].

In addition to the aforementioned selection criteria, samples were chosen that included a) as many commonly seen HLA alleles as possible (the definition of ‘common’ in this case relates to those alleles seen frequently in our tested population, typically British and Irish north-west European caucasoids); b) alleles with genomic sequences available in the IMGT/HLA Database where possible; c) alleles that have known indels; and d) one DNA sample that is well characterized and known to be homozygous for HLA-A,-B and-C. The homozygous cell line chosen was the B-LCL COX (sample AN5) and was selected for HLA typing using the SMRT sequencing method as it is known to be consanguineous and has previously undergone in-depth sequence analysis of the entire Major Histocompatibility Complex (MHC) region on chromosome 6, which includes the HLA gene family [16, 17]. This previous in-depth analysis ensured full-length HLA class I gene sequences were available for the constituent alleles. In addition, the HLA haplotype observed in this cell line has remained evolutionarily well-conserved and is one of the most frequently observed haplotypes in our tested population [18].

HLA class I amplicons were generated using primers as described for the 13th International Histocompatibility workshop [19]. This protocol enables amplification of the entire HLA Class I gene from 5´ to 3´ UTR. Fragment sizes were estimated to be 3500 bp, 3400 bp and 3450 bp for HLA-A,-B and-C respectively. The amplification method used TaKaRa LA DNA polymerase (Takara Bio Europe SAS, Saint-Germain-en-Laye, France). Agarose gel electrophoresis was used to confirm amplification and correct fragment size, as well as to check for non-specific product contamination. A 10 KB sizing marker was included to confirm size specificity (HyperLadder I; Bioline Reagents LTD, London, UK).

The HLA class I amplicons were sequenced according to Pacific Biosciences’ standard protocol for PCR amplicons greater than 3 KB in length. Briefly, HLA class I amplicons underwent quality and quantity confirmation using a Bioanalyser instrument and the Agilent DNA 12000 kit (Agilent Technologies, Santa Clara, CA, USA). As we aimed to test basic multiplexing capabilities of the SMRT DNA sequencing system, a pool of each of the HLA class I amplicons for a single sample were pooled at equimolar concentrations. After performing DNA damage and end repair, the SMRTbell adaptors were blunt-end ligated onto the PCR amplicons in the pool. Following the ligation of the adaptors, an adaptor-specific sequencing primer and enzyme were bound to the templates. For this study, sequencing was enabled by the use of the P4 enzyme and C2 chemistry. Finally, the SMRTbell templates were loaded on to MagBeads, magnetic beads that facilitate even sample loading into the SMRT Cell. DNA samples were sequenced on the PacBio RS II SMRT DNA Sequencing System with a movie capture time of 120 minutes. All stages of the sequencing process, including library preparation, SMRT Cell loading and data collection were achieved within three working days.

The DNA sequences derived from Pacific Biosciences’ SMRT sequencing technologies underwent post-processing using the SMRT analysis tool v2.1, and were assigned HLA types using Anthony Nolan in-house Bioinformatics methods. The PacBio methodology provides a number of sequences for analysis for each sample. The optimal consensus sequences for each run were selected by Anthony Nolan and Pacific Biosciences’ researchers and analysis was performed. HLA types were assigned based on identity to known sequences within the IMGT/HLA Database. Where novel sequences were reported, assignment of a HLA type was based on aligning the novel consensus sequence at both the cDNA, gDNA and protein level, to identify the nearest known HLA allele.

Sanger sequencing was used to determine the accuracy of regions of DNA sequence obtained from SMRT sequencing that either differed to the existing genomic sequences for the expected allele, or if no genomic sequence were available, differed to that seen in the closest matching allele. SBT was enabled using BigDye Terminator sequencing kit V3.1 (Applied Biosystems, Foster City, California, USA) and utilised primers designed in-house. Fragments were sequenced on an ABI 3730XL Genetic Analyser (Applied Biosystems, Foster City, California, USA). As the majority of the tested samples were heterozygous for each of the HLA class I loci, generic PCR and SBT was not sufficient in some cases to enable confirmation of discrepancies. For these samples, cloning of full-length HLA gene PCR amplicons was used to allow separation of the two alleles. HLA class I PCR products were cloned using the Zero Blunt TOPO cloning kit (Life Technologies, Paisley, UK) before targeted sequencing as previously described.

## Results

Seven DNA samples were selected for SMRT DNA sequencing based on a set of defined inclusion criteria which included different starting material from which the DNA was extracted and the inclusion of as many commonly seen HLA class I alleles as was feasible. Each of the seven samples tested were able to generate sufficient quality sequence data to create a consensus sequence for all of the alleles expected. Variation was seen in the number of sub-reads achieved for each allele due to allelic imbalance that occurs during PCR amplification that is not routinely detected with HLA typing strategies that do not allow sequencing of single gene sequences in isolation. Despite these imbalances, the minimum depth of coverage was still in excess of 150x (median 462.5; range 154–2931), that is there were in excess of 150 sub-reads of sufficient quality for each allele once subjected to the quality checks in the post-processing stage of SMRT data analysis, that could be used to generate a consensus sequence (Table 1). 100% of the total number of consensus sequences generated achieved a mean Quality Value (QV) of over 70 (mean QV 74.079, range 71.937–80).

**Table 1:** Depth of coverage achieved using SMRT sequencing.

ID	HLA-A		HLA-B		HLA-C	
	Allele	Number of sub-reads	Allele	Number of sub-reads	Allele	Number of sub-reads
**AN1**	A*03:01	303	B*07:02	841	C*05:01	569
	A*11:01	385	B*44:02	780	C*07:02	726
**AN2**	A*25:01/02	353	B*15:01	817	C*03:03	514
	A*68:01:02	184	B*18:01	583	C*12:03	422
**AN3**	A*26:01	238	B*14:01	1263	C*02:02	282
	A*31:01:02	371	B*27:05:02	498	C*08:02	162
**AN4**	A*03:01	300	B*27:05:18	197	C*01:02	213
	A*32:01	799	B*35:01	836	C*04:01	584
**AN5**	A*01:01	1477	B*08:01	2134	C*07:01	2931
**AN6**	A*02:01	516	B*52:01	247	C*07:01	278
			B*73:01	427	C*15:05	156
**AN7**	A*23:01	349	B*42:01	327	C*06:02	1080
	A*24:02	313	B*50:01	1390	C*17:01	1840

A comparison of the HLA typing results expected based on HLA class I typing by Anthony Nolan and that obtained through SMRT sequencing can be found in Table 2. Samples that were thought to be homozygous at a particular locus were expected to generate a single consensus sequence. Alleles thought to be the same but observed in different individuals (for example, HLA-A*03:01:01:01 in samples AN1 and AN4) were considered as different consensus sequences. Therefore, a total of 38 possible consensus sequences were expected.

**Table 2:** A comparison of expected HLA types, as typed by Anthony Nolan, with those generated by the Single Molecule Real-Time (SMRT) DNA Sequencing method.

ID	DNA source	Results group*	HLA-A allele 1	HLA-A allele 2	HLA-B allele 1	HLA-B allele 2	HLA-C allele 1	HLA-C allele 2
AN1	Saliva	AN	*03	*11	*07	*44	*05	*07
		SMRT	*03:01:01:01	*11:01:01	*07:02:01	*44:02:01:01	*05:01:01:02	*07:02:01:03
AN2	Blood	AN	*25:01/02	*68	*15	*18	*03:03	*12:03
		SMRT	*25:01:01	*68:01:02:02	*15:01:01:01	*18:01:01:02	*03:03:01	*12:03:01:01
AN3	Saliva	AN	*26:01:01	*31:01:02	*14:01	*27:05/13	*02	*08
		SMRT	*26:01:01	*31:01:02	*14:*01*:*01*	*27:05:02	*02:02:02:02	*08:02:01:02
AN4	Blood	AN	*03	*32:01	*27:05:18	*35:01	*01	*04
		SMRT	*03:01:01:01	*32:01:01	*27:*05*:*18*	*35:01:01:02	*01:02:01	*04:01:01:01
AN5	B-LCL	AN	*01:01:01:01	*01:01:01:01	*08:01:01	*08:01:01	*07:01:01:01	*07:01:01:01
		SMRT	*01:01:01:01	*01:01:01:01	*08:01:01	*08:01:01	*07:01:01:01	*07:01:01:01
AN6	B-LCL	AN	*02:01	*02:01	*52:01:01	*73:01	*07:01	*15:05
		SMRT	*02:01:01:01	*02:01:01:01	*52:01:01:03	*73:01	*07:01:01:01	*15:*05*:*01*
AN7	B-LCL	AN	*23:01	*24:02:01:01	*50:01	*42:01	*06:02	*17:01
		SMRT	*23:01:01	*24:02:01:01	*50:01:01	*42:01:01	*06:02:01:02	*17:01:01:02

HLA alleles in bold highlight novel alleles, genomic sequence corrections or genomic sequence extensions.

* AN—Anthony Nolan typing data as generated by Luminex LABType SSO typing kits (One Lambda, CA, USA), Sequencing-based typing and/or PCR using Sequence specific primers (PCR-SSP).

SMRT—Single Molecule Real-Time DNA sequencing method from Pacific Biosciences

Thirty of these 38 possible HLA consensus sequences immediately showed complete identity with reference sequences available in the IMGT/HLA Database (Table 2). Both alleles for each of the three HLA class I loci were accurately called in three samples, AN1, AN5 and AN7. SMRT sequencing of sample AN5 correctly identified this sample to be homozygous for all three HLA class I loci tested. Unexpectedly, an additional HLA gene sequence was also identified in this sample. Sequences corresponding to the HLA pseudogene allele HLA-H*02:01:01:01 were identified, although the numbers of reads were low (n = 12). Despite the suboptimal number of reads, the HLA-H sequences were correctly called for this sample. The reason for this non-specific product is due to the co-amplification of HLA-H in the reactions for another class I product, presumably HLA-A due to sequence similarities between the two genes. Samples AN6 and AN7 were included in this test cohort as they contain alleles that have notable non-coding deletions in their genomic DNA (gDNA) sequences (B*73:01 and C*17:01). SMRT sequencing methods were able to generate consensus sequences that accurately identified these two alleles.

Four consensus sequences generated with SMRT DNA sequencing methods matched alleles that only had either partial gene or Coding DNA Sequences (CDS) available in the IMGT/HLA Database. As described previously, HLA types were assigned to the consensus sequences by comparison to CDS sequences available on all HLA alleles as well as with genomic sequences of closest related alleles (Table 3). Data for sample AN6 (HLA-C*15:05:01 expected) was identical to the reference genomic sequence used as a comparison (HLA-C*15:05:02) except for the single nucleotide difference in exon 1 that differentiates the two alleles (gDNA 24T>C). Samples AN3 (HLA-B*14:01:01 expected) and AN4 (HLA-B*27:05:18 expected) showed sequence variation in intron sequences in addition to those that define the differences between the observed allele and that to which it was being compared. Sanger sequencing was used to confirm allele identity (AN6) or to confirm the existence of novel non-coding variants (AN3 and AN4). All positions tested matched those generated with SMRT sequencing technology confirming the accuracy of the method. Data for the second HLA-B allele in sample AN3 initially suggested an intron 5 variant of HLA-B*27:05:02 (gDNA 2086C>T). Sanger sequencing of the region of interest confirmed the nucleotide substitution when compared to the existing genomic reference sequence. An analysis of all available intron 5 sequences for HLA-B*27 alleles showed all other alleles had the variant base at the queried position (2086T), possibly suggesting that there was an error in the original sequence submitted to the IMGT/HLA Database. The original DNA source used to generate the HLA-B*27:05:02 genomic sequence was identified and re-sequenced. The data confirmed that there was an error in the original genomic sequence and that the consensus sequence generated by SMRT DNA sequencing was correct. These novel genomic HLA sequences have been submitted to the IMGT/HLA Database as extensions or corrections to existing alleles (Table 3).

**Table 3:** Extensions and corrections to known HLA alleles identified in PacBio results.

Sample	Expected Allele	Allele used for genomic sequence comparisons	Novel/ differentiating variants	Sequence type	EMBL Accession numbers
AN3	B*14:01:01	B*14:02:01	Intron 2 G>T gDNA 665	Extension	HG794368
AN3	B*27:05:02	B*27:05:02	Intron 5 C>T gDNA 2086	Correction	HG794364
AN4	B*27:05:18	B*27:05:02	Exon 2 C>T gDNA 269;	Extension	HG530757
AN6	C*15:05:01	C*15:05:02	None	Extension	HG794367

Four of the 38 tested alleles showed novel genomic HLA sequences when compared to the expected sequences (Table 4); AN2 (HLA-A*68 variant), AN6 (HLA-B*52 variant) and AN3 (two HLA-C variants, C*02 and C*08). Sanger SBT of the regions of interest for each of the four alleles confirmed the variant bases, and thus the novel alleles identified using SMRT sequencing. These novel genomic sequences have been submitted to the IMGT/HLA Database and have been officially named according to the WHO Nomenclature Committee for Factors of the HLA System (Table 4) [3].

**Table 4:** Anomalies observed in the PacBio SMRT consensus sequences as compared to the expected allele.

Sample	Expected Allele	Variants	Sequence Confirmed	New allele name	EMBL Accession number
AN2	A*68:01:02	Intron 7 G>A gDNA 2770	Confirmed,new variant	A*68:01:02:02	HG794362
AN3	C*02:02:02	Intron 5 T>C gDNA 2487	Confirmed,new variant	C*02:02:02:02	HG794365
AN3	C*08:02:01	Intron 3 A>G gDNA 1338	Confirmed new variant	C*08:02:01:02	HG794366
AN6	B*52:01:01:02	5´ UTR C>A gDNA -180	Confirmed new variant	B*52:01:01:03	HG794363

When Sanger sequencing confirmations of novel genomic sequences were included, the final analysis showed absolute concordance between the consensus sequences generated with Pacific Biosciences’ SMRT DNA sequencing method and the expected/observed alleles.

A common concern with all sequencing-based typing methods (second generation and in some cases, Sanger sequencing) is the accuracy of the technology to determine the correct number of consecutive nucleotides within homopolymer regions. This is of utmost importance in HLA testing as a single nucleotide insertion/deletion will change the HLA type of an individual, which can have serious clinical consequences. In order to assess the accuracy of SMRT DNA sequencing technology, we determined the number of homopolymer regions present in each of the 38 HLA sequences generated that consisted of five or more nucleotides (Table 5). The total number of bases sequenced was 130117 bp within which 487 homopolymer regions were identified. The most frequently observed nucleotide repeat regions were 5-mers, which occurred multiple times for each of the four nucleotides (range 12–209 times). The longest homopolymer region found in the tested alleles was a 9-mer; this occurred 13 times but only for the T nucleotide. Noticeably fewer homopolymer regions were observed for the A, C and G nucleotides, particularly in 7-, 8- and 9-mers. In all cases, SMRT DNA sequencing methodology accurately determined the correct number of nucleotides present in each allele. Additionally, 99.354% of the homopolymer regions had a mean QV of 70 or more across the homopolymer region (mean QV 74.084; range 64.286–80). Details on individual QV data for each of the 38 HLA sequences generated can be found in the supplementary information (S1 Table).

**Table 5:** Homopolymer count in 38 PacBio sequences (total length: 130117 bp).

	Nucleotide			
Homopolymer count	A	C	G	T
5-mers	12	160	209	25
6-mers	0	29	16	0
7-mers	0	7	2	10
8-mers	0	0	2	2
9-mers	0	0	0	13

## Discussion

Next generation sequencing technologies have offered the first feasible laboratory-based solution to the problem of phasing the complex polymorphisms seen in the HLA gene family. Limitations in read length have meant that a shotgun approach has to be applied, with multiple fragments covering an entire region of interest being necessary [10, 12, 20–24]. The SMRT DNA sequencing method from Pacific Biosciences has overcome the need to sequence multiple overlapping fragments allowing sequencing of a single fragment in excess of 20 kb in one sequencing reaction. The implications of this technology in the field of HLA typing could be enormous, allowing for true allelic HLA typing in a single experimental set-up and making redundant the need for multiple experiments on different typing platforms, cross-referencing of results and/or the need for re-sequencing using an allele specific protocol. We have described here the results of a feasibility study which shows that whole HLA class I gene sequencing is possible using the SMRT DNA sequencing platform. The sequence data generated was high quality and allowed for accurate allele calling. In addition, all stages of the experimental set-up were completed within three working days and sequence data were captured over 120 minutes. In combination, these factors make the SMRT DNA sequencing method amenable for use in a high-throughput HLA typing laboratory.

The primary aim in testing this methodology was to determine whether accurate genomic consensus sequences could be generated using our current in-house protocol for full gene HLA class I amplification and with our DNA samples using SMRT sequencing technology. Our findings have confirmed that our blood and saliva specimens and subsequent DNA extraction procedures are suitable for the isolation of high molecular weight genomic DNA, an essential prerequisite for the PCR amplification of HLA whole gene amplicons. The PCR primers and amplification conditions led to specific amplification of the genes of interest, namely HLA-A,-B and-C. There was minimal co-amplification of HLA-H, most likely with HLA-A primers, but this did not have a detrimental effect on allele calling. Some allelic imbalance was observed in the data generated for HLA-B. The most likely explanations for this observation are either that SMRT DNA sequencing is a more sensitive methodology and is therefore more likely to identify imbalance in the PCR which is not seen in SBT, or that possible nucleotide differences between the primer and allele sequences caused inefficient or inhibited binding.

Differences between the numbers of reads seen for each locus of a single sample were also observed. A potential reason for these differences is that there was imbalance during the equimolar pooling stages, with some loci being over or under represented. Additionally, the kit used to quantify the PCR amplicons prior to equimolar pooling is limited to quantifying samples within a range of 0.5–50 ng/μl. As all amplicons in this experiment were of concentrations towards the upper limits of the kit, it is possible that the sizing and quantification values were affected, which consequently affected the volumes required for equimolar pooling and causing the imbalance between loci. The use of single molecule sequencing methodologies is challenging our previous perceptions of what constitutes ‘good’ or ‘successful’ PCR amplifications, with significantly lower quantities of amplicons required for most processes.

Despite imbalance issues, significant depth of coverage was achieved for all alleles that were sequenced and allowed for accurate HLA allele assignment. Future experiments where the extent of multiplexing different DNA samples or HLA loci is tested should consider the affect of allelic imbalance on the depth of coverage achievable, although these issues should be easily rectified with additional amplification optimisation. Additionally, future experiments should either take final concentrations of samples and quantification kit limitations into consideration before proceeding with the sequencing experiment, or alternatively, PCR conditions altered to allow for lower quantities of amplicon to be generated.

The concentration of amplicons for all three class I loci was sufficient for pooling at equimolar levels prior to library preparation. The multiplexing of the three amplicons from a single sample in a designated SMRT cell allowed for 150x read depth for all alleles present with the resultant sequence reads being successfully aligned and assigned to the relevant HLA class I genes with analysis software. The amplicon lengths were similar enough to negate the potential problem of loading bias towards smaller PCR products in a pool when dispensed into SMRT Cells. The generated sequence exhibited complete coverage from the sites of the PCR primers. Depending on the loci and alleles present, this was inclusive of the terminal 300 bp in the 5´UTR, exons, introns and the leading 200 bp in the 3´UTR.

The quality of the HLA class I genomic sequences generated can partly be confirmed by the high percentage of those reaching QV70, in some cases higher, but also by the accurate assignment of HLA types to these sequences. Of particular interest was the accuracy of the data produced for the homopolymer regions present in the different alleles due to the known cross-platform problem of enzymes incurring slippage when sequencing through long stretches of a single continuous base. In all cases, SMRT DNA sequencing technology was able to call the correct number of bases for each allele. The longest homopolymer region sequenced here was a 9-mer and although this was seen multiple times, only 9-mers of the T nucleotide were observed. Thus it remains to be seen whether the technology can adequately sequence through longer homopolymer regions and whether different bases introduce other problems.

The accuracy of the methodology for sequencing the tested samples was substantiated by the correct identification of novel HLA class I alleles, each of which was separately confirmed using Sanger-based sequencing methods. The high number of novel alleles found in this small test cohort (4/38 sequences; 10.5%) highlights the extensive polymorphism seen in the HLA genes outside of the routinely typed exons, much of which may as yet be unknown. As previously stated, most histocompatibility laboratories would like to be able to generate allele-level resolution for all samples processed, but this is often unattainable due to financial, time and experimental constraints. SMRT DNA sequencing technology could offer a resolution to these issues, providing sequences for ultra-high resolution HLA typing in a single sequencing reaction and being achievable in less time than it would take using current methodologies.

The down-stream uses of HLA typing data are varied and include assessing compatibility between donors and recipients prior to transplantation, drug hypersensitivity and disease associations. The potential impact of using SMRT DNA sequencing in the future to generate such high-resolution HLA typing on many of these areas of medicine are likely to be considerable. For example, high resolution HLA typing has been shown to significantly improve outcome when stem cell transplant recipients and their unrelated donors are matched for both alleles at five of the classical HLA loci (HLA-A,-B,-C,-DRB1 and-DQB1, a 10/10 match) [25–28], as it is thought that disparity at these important compatibility loci can contribute to complications such as graft-versus-host disease and consequently, to mortality. SMRT DNA sequencing has the potential to detect previously unidentified polymorphisms in regions of the HLA genes that could be significantly contributing to these complications. This could ultimately result in considerable improvement in survival rates post transplant.

Currently many histocompatibility laboratory regulatory bodies are defining the standards that will be necessary for clinical typing and reporting of HLA types by various sequencing platforms, particularly regarding the minimum depth of coverage required. At this time, no clinical governance has been established. The depth of sequence coverage described to date in HLA studies that have utilised next generation sequencing methods has varied [10, 12, 20–24]. Here we have demonstrated a minimum of 150x depth of coverage for each of the alleles tested, with the added advantage that each of the sub-reads are full genomic sequences. However, as this was a feasibility study, we have not tested the maximum capabilities of the SMRT DNA sequencing method, with a maximum of six individual amplicons (two different alleles per HLA gene; three HLA genes per DNA sample tested) being sequenced on a single SMRT Cell. In order for this technology to be economically and practically viable for use in our clinical laboratories, the degree of multiplexing must be significantly higher. What effect this would have on the depth of coverage achievable for a single allele is yet to be determined, but it is reasonable to assume that it would be notably lower than experienced in this study. Thus, the potential of SMRT sequencing for routine HLA typing at this current time will in some part be dictated by the cost per sample, but also by the requirements of the histocompatibility laboratory regulatory bodies. Preliminary data from our group suggests that multiplexing 48 samples for three HLA class I genes is possible and produces accurate typing results, suggesting that this technology is viable for use in a high-throughput clinical laboratory (unpublished data).

The number of DNA samples tested here were low although multiple genes were sequenced for each sample. It is important that larger and more diverse cohorts of DNA samples are sequenced using SMRT DNA technology to confirm suitability for HLA typing. Future studies should also test the maximum multiplexing capabilities of the SMRT sequencing system, both with increased numbers of samples and the number of HLA loci included per SMRT Cell. It also remains to be seen whether accurate and high-quality HLA class II consensus sequences can be generated on this platform, which would be necessary for clinical use of this technology.

This method offers a realistic solution to the issues encountered in clinical HLA typing and has the potential to significantly improve clinical prognoses.

## Supporting Information

S1 Table Accession numbers and QV values of all HLA genomic sequences generated using SMRT DNA sequencing method and submitted to EMBL. (DOCX)
