# Supplementary material for: HLA Typing for the Next Generation
Source: PLoS One. 2015 May 27;10(5):e0127153. doi: 10.1371/journal.pone.0127153 (PMC4446346; doi:10.1371/journal.pone.0127153)
Supplement: S1 Table — (DOCX) [file pone.0127153.s001.docx]

**Supplemental data:**

**S1 Table: Accession numbers and QV values of all HLA genomic sequences generated using SMRT DNA sequencing method and submitted to EMBL.**

| **HLA Allele** | **Sample** | **Comment** | **Accession number** | **Average QV score** |
| --- | --- | --- | --- | --- |
| A*03:01:01:01 | AN1 | Confirmation | HG794390 | 72 |
| A*11:01:01:01 | AN1 | Confirmation | HG794391 | 72 |
| A*25:01:01 | AN2 | Confirmation | HG794369 | 72 |
| A*68:01:02:02 | AN2 | Novel allele | HG794362 | 72 |
| A*26:01:01 | AN3 | Confirmation | HG794395 | 72 |
| A*31:01:02 | AN3 | Confirmation | HG794385 | 72 |
| A*03:01:01:01 | AN4 | Confirmation | HG794396 | 80 |
| A*32:01:01 | AN4 | Confirmation | HG794386 | 80 |
| A*01:01:01:01 | AN5 | Confirmation | HG794373 | 72 |
| A*02:01:01:01 | AN6 | Confirmation | HG794376 | 72 |
| A*23:01:01 | AN7 | Confirmation | HG794379 | 72 |
| A*24:02:01:01 | AN7 | Confirmation | HG794380 | 72 |
| B*07:02:01 | AN1 | Confirmation | HG794392 | 72 |
| B*44:02:01:01 | AN1 | Confirmation | HG794398 | 72 |
| B*15:01:01:01 | AN2 | Confirmation | HG794370 | 72 |
| B*18:01:01:02 | AN2 | Confirmation | HG794397 | 72 |
| B*14:01:01 | AN3 | Genomic sequence extension | HG794368 | 72 |
| B*27:05:02 | AN3 | Genomic sequence correction | HG794364 | 72 |
| B*27:05:18 | AN4 | Confirmation | HG530757 | 79.99 |
| B*35:01:01:02 | AN4 | Confirmation | HG794387 | 80 |
| B*08:01:01 | AN5 | Confirmation | HG794374 | 72 |
| B*52:01:01:03 | AN6 | Novel allele | HG794363 | 71.99 |
| B*73:01 | AN6 | Confirmation | HG794377 | 72 |
| B*42:01 | AN7 | Confirmation | HG794381 | 72 |
| B*50:01 | AN7 | Confirmation | HG794382 | 72 |
| C*05:01:01:02 | AN1 | Confirmation | HG794394 | 72 |
| C*07:02:01:03 | AN1 | Confirmation | HG794393 | 72 |
| C*03:03:01 | AN2 | Confirmation | HG794371 | 72 |
| C*12:03:01:01 | AN2 | Confirmation | HG794372 | 72 |
| C*02:02:02:02 | AN3 | Novel allele | HG794365 | 72 |
| C*08:02:01:02 | AN3 | Novel allele | HG794366 | 71.99 |
| C*01:02:01 | AN4 | Confirmation | HG794388 | 80 |
| C*04:01:01:01 | AN4 | Confirmation | HG794389 | 80 |
| C*07:01:01:01 | AN5 | Confirmation | HG794375 | 72 |
| C*07:01:01:01 | AN6 | Confirmation | HG794378 | 72 |
| C*15:05:01 | AN6 | Genomic sequence extension | HG794367 | 72 |
| C*06:02:01:02 | AN7 | Confirmation | HG794383 | 72 |
| C*17:01:01:02 | AN7 | Confirmation | HG794384 | 72 |
